# Neuroglia in cognitive reserve

**DOI:** 10.1038/s41380-024-02644-z

**Published:** 2024-07-02

**Authors:** Alexei Verkhratsky, Robert Zorec

**Affiliations:** 1 Faculty of Biology, Medicine and Health, The University of Manchester, Manchester, M13 9PT UK; 2 Department of Neurosciences, University of the Basque Country, 48940 Leioa, Bizkaia Spain; 3 IKERBASQUE Basque Foundation for Science, Bilbao, Spain; 4 University of Ljubljana, Institute of Pathophysiology, Laboratory of Neuroendocrinology and Molecular Cell Physiology, Zaloška cesta 4, SI-1000 Ljubljana, Slovenia; 5 Celica, BIOMEDICAL, Technology Park 24, 1000 Ljubljana, Slovenia

**Keywords:** Biological sciences, Neuroscience

## Abstract

The concept of cognitive reserve was born to account for the disjunction between the objective extent of brain damage in pathology and its clinical and intellectual outcome. The cognitive reserve comprises structural (brain reserve) and functional (brain maintenance, resilience, compensation) aspects of the nervous tissue reflecting exposome-driven life-long plasticity, which defines the ability of the brain to withstand aging and pathology. The mechanistic background of this concept was primarily focused on adaptive changes in neurones and neuronal networks. We present arguments favoring the more inclusive view, positing that neuroglia are fundamental for defining the cognitive reserve through homeostatic, neuroprotective, and neurodegenerative mechanisms. Neuroglia are critical for the life-long shaping of synaptically connected neuronal circuits as well as the brain connectome thus defining cognitive reserve. Neuroglial homeostatic and protective physiological responses define brain maintenance and resilience, while neuroglia regenerative capabilities are critical for brain compensation in pathology. Targeting neuroglia may represent an untrodden path for prolonging cognitive longevity.

## The concept of cognitive reserve

It is a truth universally acknowledged, that the functional consequences of brain damage are highly individual and are out of joint with the degree of the injury. The very same structural damage to the brain leads to widely different neurological and cognitive outcomes in different patients. This applies to all diseases of the central nervous system (CNS), including trauma (mechanical, toxic, or autoimmune), infection (local or systemic), or ischaemia/stroke. Similarly, individuals display widely different susceptibility to chronic diseases, as in stress-induced psychiatric disorders and in age-dependent neurodegeneration. It is the disjunction between structural brain damage and its clinical outcome, both cognitive and neurological, that lies behind the concept of cognitive reserve formalised by Yaakov Stern at the beginning of the third millennium [1–5].

Clinical and cognitive manifestations of Alzheimer’s disease (AD), the most commonly diagnosed form of dementia, highlight substantial individual differences. It is undeniable that AD stems from a complex of specific genetic and molecular factors, while accumulated environment-induced alterations significantly contribute to the disease progression and cognitive deficit [6, 7]. Although early studies established a degree of correlation between mean plaque count, a hallmark of neurodegeneration, in the post-mortem brains of patients and their cognitive impairment determined antemortem, the same studies revealed individual cases with high plaque loads but preserved memory and intelligence [8]. Similarly, a study of elderly (aged 70–103 years) population found that around one-third of aged people whose neuropsychological score prior to death was unimpaired met full pathohistological post-mortem criteria for AD [9]. These findings emphasised individual differences in the capability of the brain to withstand insults and provide for functional compensation. Thus the brains were deemed different, reflecting that brain development is determined by both genetic and acquired influences; with functional training, education, intellectual and sociocultural engagement improving, through neural plasticity and systemic factors, brain resilience to aging and neuropathology [10–12]. At the same time, diseases and stresses experienced by the individual during life span lead to an accumulation of pathological burden, increasing brain vulnerability to incoming insults. Thus life experience may have both beneficial and adverse effects on the ability of the brain to withstand pathology through positively or negatively modulating the cognitive reserve.

## The complexity of the cognitive reserve

Conceptually, cognitive reserve defines an individual property of the brain to maintain cognitive performance above expected age- or disease-inflicted damage. Fundamentally, cognitive reserve is the result of the history of the interaction of every given brain with life-long exposure to environmental factors (commonly known as exposome) leading to beneficial (life-long learning, plasticity) or detrimental (accumulation of pathological changes) modifications. These interactions also depend on the genes, which encode individual brains. These modifications are specific to the nervous tissue as well as systemic, both being in the most intimate interrelation.

The cognitive reserve as it is currently considered [4] includes (i) the brain reserve, (ii) the brain maintenance, (iii) the brain resilience (this concept was initially developed in psychiatry as a measure of ability to tolerate environmental stress without pathological changes [13, 14]) and (iv) the brain compensation reflecting the regenerative capacity of the brain, which defines the postlesional neurological, structural, and functional recovery.

### Brain reserve: a passive principle

Brain reserve (defined initially as neural or functional reserve [15]), was considered from purely anatomical view as a resource determined by the size and/or structural properties of every given brain at the time of the insult. Initially, the brain reserve was linked to the brain size (incidentally, people with larger brains have lesser prevalence of dementia [16, 17]) or the number of neurones and synapses; the larger are these numbers and the total brain size the more damage can the individual brain absorb, exhibiting higher reserve [15, 18]. The brain reserve therefore is a ‘passive’ principle which defines the ‘threshold’ of the neuroanatomical structure which has to be reached for cognitive impairments to become clinically apparent. At the same time, the anatomical architecture of the brain and neuronal connectivity are sculpted by life-long plasticity.

### Cognitive reserve: an active principle

The cognitive reserve represents a more active principle, reflecting adaptations attained in the course of life. Accordingly, the brain structure is being continually remodelled through learning and memory as well as in response to insults encountered through life. Thus, cognitive reserve is a property of every individual; it reflects the life-long interaction of genetic factors with the environment, accumulated plastic (morphological and functional) changes of the nervous tissue as well as pathological damages accrued throughout life. At the moment of injury, the life-long plastic experience, which sculpts the brain structure, as well as the functional state of defensive and homeostatic systems, determines the susceptibility to the disease as well as the level of cognitive decline and/or recovery. These factors also define brain resilience which is the capacity of the brain to withstand the insult without developing the pathology.

Cognitive reserve includes other components defining the overall capacity of reserve and resilience, represented by brain maintenance and brain compensation. The brain maintenance in particular determines physiological brain ageing and cognitive longevity by employing various mechanisms preserving the brain physiology. Finally, brain compensation refers to multiple protective mechanisms aimed at the repair and regeneration of damaged neuronal circuits or damaged nervous tissue to restore brain health and cognitive capacity.

## Cellular mechanisms of the cognitive reserve: the role for neuroglia

For almost a century the understanding of how the nervous system functions in health and disease focused on neurones. Therefore, it is not surprising that the neuronocentric view in neurophysiology, neurology, psychology, and psychiatry also earmarked neurones as the site of the cognitive reserve [4, 5]. The nervous tissue however is made not only of neurones, it includes non-neuronal elements, represented by neuroglia and cells of brain vessels [19]. Evolution of the nervous system progressed through the division of functions, segregating neurones, responsible for rapid information processing and transfer, and neuroglia, responsible for homeostasis and defence of the nervous tissue [20]. All cellular elements of the nervous tissue (neurones, neuroglia, and cells of vasculature) operate in the closest concert, being highly interdependent and forming the active milieu of the brain, characterised by elaborated feedback signalling [21]. Contributions of different cell types are nonetheless different being determined by specific sets of genes defining cellular functions executed in distinct time domains [22]. The role for neuronal plasticity which shapes, through learning, synaptic contacts, and connectome in defining the cognitive reserve is universally acknowledged [23]. The maintenance of massive brain vascularisation (the total length of human brain blood vessels is around 600 km [24]) is similarly critical for cognitive reserve. Both endothelial cells (the human brain contains 5–10 billions of them) and pericytes undergo complex alterations in the course of life and contribute to the age-dependent remodelling of the brain vasculature which defines blood supply and hence metabolic wellbeing of the nervous tissue. In particular, pericytes are coming to the spotlight because of their roles in brain compensation and repair [25]. In this perspective, however, we focus on the role of neuroglia, the homeostatic and defensive cells of the nervous system, which are fundamental for life-long adaptation of the nervous tissue.

**Fig. 1:**
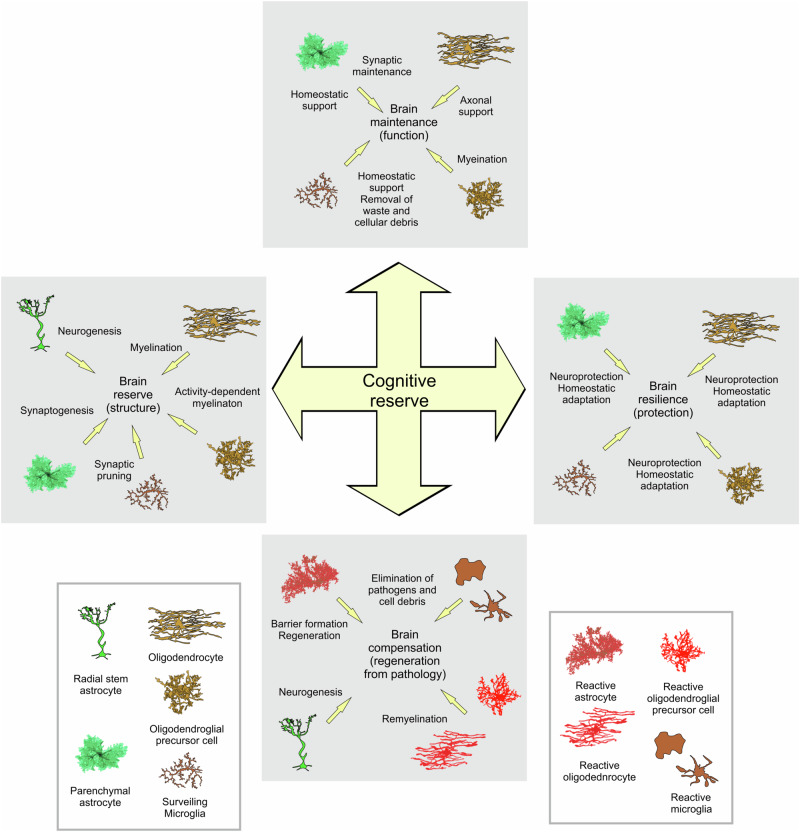
Neuroglia contribution to the cognitive reserve. Neuroglial cells are fundamental for all aspects of cognitive reserve. *Brain reserve*. Neuroglia working in concert with neurones define the brain structure and shapes its cytoarchitecture. Radial glia (prenatally) and stem radial astrocytes (postnatally) provide for neurogenesis. Astrocytes, through secretion of multiple factors such as trombospondins, hevins, glypicans, and cholesterol stimulate synaptogenesis, control synaptic maturation, and provide material for the adaptive remodelling of neuronal membranes. Microglia thorough synaptic pruning and trogocytosis shape synaptically connected neuronal ensembles, while oligodendroglial cells support connectome and provide for activity-dependent life-long myelination. *Brain maintenance and brain resilience*. Neuroglial cells through multiple molecular cascades support CNS homeostasis and define brain resilience through numerous protective pathways. *Brain compensation*. Neuroglia are central element of brain defense and regeneration; reactive glial cells erect perilesional barriers, remove pathogens and cellular debris, and orchestrate regeneration.

Neuroglial cells (which, in the CNS are represented by astroglia, oligodendroglia, and microglia) are endowed with intricate molecular cascades controlling the nervous tissue environment in health and responding to the disease conditions through mounting defensive responses. Thus neuroglia support physiological neuroplasticity at multiple levels; neuroglial cells also define the progression and outcome of all neurological diseases [19, 26–28]. Similarly, neuroglia, acting through the cell-specific mechanisms [29], contribute and, to a large extent, define cognitive reserve (Fig. 1).

### Neuroglia and the brain reserve

As mentioned before, the brain reserve is defined by a number of neurones and interneuronal connections formed by axonal projections (the connectome) and synapses. All these are regulated by neuroglia. Embryonic neurogenesis is the function of radial glia, whereas adult neurogenesis is supported by radial stem astrocytes (also known as neural stem cells, but exhibiting all major properties of astroglia [30–32]). Synaptic connectivity is regulated by astrocytes, which secrete numerous factors regulating synaptogenesis, synaptic maturation, and synaptic extinction [33–38], as well as by microglia, which remove redundant, silent or malfunctional synapses through synaptic pruning, thus shaping neuronal ensembles [39–42]. Brain-wide connectome is supported by oligodendroglia [19, 43], which is also responsible for activity-dependent myelination [44, 45]. White matter occupies ~50% of the adult human brain, in comparison with ~10% in rodents, and is one of the main determinants of the human brain computing power and cognitive abilities [19].

### Neuroglia and the brain maintenance

The brain maintenance keeps all in optimal near-equilibrium steady state, defined by Claude Bernard as a stability of the *milieu interior* [46] and by Walter Cannon as homeostasis [47]. Homeostasis of the CNS (as well as in all other tissues, organs, and indeed the whole organism) is not static or fixed, but is achieved through constant adjustments to environmental challenges, termed adaptive homeostasis [48], or allostasis (*αλλο −* variable and *στασισ* - standing still - that is ‘remaining stable by being variable’ or ‘maintaining homeostasis through change’ [49]). Astroglia, the main homeostatic cells of the CNS, play the dominant role in brain maintenance. Astrocytes are central for the dynamic regulation of the ionic composition of the CNS interstitium, the ionostasis, through a wide array of dedicated pumps and transporters; astrocytes control all major biological ions (Na^+^, Ca^2+^, K^+^, and Cl^−^ [50–52]) and trace metals indispensable for CNS function [53]. Astrocytes are key elements of neurotransmission, through neurotransmitter clearance (astrocytes express transporters removing glutamate, GABA, catecholamines, and adenosine), neurotransmitter catabolism (astrocytes convert glutamate to glutamine and degrade catecholamines and adenosine), and they supply neurones with obligatory neurotransmitter precursors such as glutamine or L-serine [19]. Astrocytes mediate neuroprotection through multiple pathways; in particular, astrocytes represent the main anti-oxidant system of the CNS [54–56]. Astrocytes contain glycogen and glycogenolysis results in the production of L-lactate, which can be used as fuel by neurones [57–59]. Similarly, oligodendrocytes support axons [60], while oligodendroglial precursor cells (OPCs) provide for life-long remyelination and de novo activity-dependent myelination as well as contribute to overall CNS homeostasis [61]. Finally, microglia are involved in debris removal, ongoing repairs, and maintenance of the immunological homeostasis of the nervous tissue [62].

### Neuroglia and the brain resilience

The brain resilience to insults depends on the ability of the nervous tissue to absorb certain amount of damage without developing pathology. The mechanisms of resilience are obscure, although the role of neuroglia is supposedly substantial. Every insult disturbs homeostasis and these are the neuroglia that by mounting homeostatic response counteract environmental challenges. When such response is compromised the pathology ensues. In chronic stress, for example, astrocytes demonstrate prominent atrophy, which arguably underlies loss of function and aberrant neurotransmission leading to depressive behaviours [63, 64]. At the same time in some animals subjected to the same stress astrocytes remain unperturbed, which correlates with resilience to depression (own unpublished observations).

### Neuroglia and the brain compensation

Neuroglia represent the principal defensive system of the CNS. Neuroglial cells actively respond to pathology by mounting reactive gliosis, an evolutionary conserved program of glial defence [26, 28, 65]. Reactive gliosis is complex, context-, and disease-specific. Brain trauma (mechanical, infectious, ischaemic or autoimmune) triggers proliferative anysomorphic gliosis, characterised by the proliferation and accumulation of astrocytes, microglia, and OPCs close to the lesion to form the protective glial barrier. In addition, glial cells undergo substantial biochemical and morphological remodelling (some microglia turn into phagocyting macrophage-like cells clearing the debris inside the lesion core; astrocytes lose their complex arborisations and become barrier-reactive astrocytes [19, 28, 65, 66]). Glial barriers effectively protect surrounding nervous tissue, assist wound closure, and are indispensable for postlesional regeneration [67, 68]. Regeneration of the brain is also supported by neurogenesis which is provided by radial stem astrocytes [69, 70]. In chronic pathologies, including AD, astrocytes undergo isomorphic, non-proliferative gliosis; in AD reactive astrocytes together with reactive microglia surround senile plaques thus limiting neuronal damage [71–73]. This protective effect is diminishing with age and AD progression; glial paralysis at the advanced stages of the disease facilities neuronal death that translates into clinical dementia [74]. Finally, OPCs in various forms of pathologies act as a source for regenerative remyelination [61]. All in all, neuroglial cells are central for brain compensation.

### Neuroglia, ageing and age-associated cognitive decline

Ageing is associated with cognitive decline and ageing is the main risk factor for neurodegenerative disorders. Physiological ageing is characterised by relative preservation of neuronal numbers [75], and a significant loss of white matter [76] associated with a decrease in number of oligodendrocytes and proliferative arrest of OPCs [61]. Both astrocytes and microglia demonstrate significant atrophy in the aged brain, which translates into decreased homeostatic support and defensive capacity [77, 78]. Ageing is also associated with tau astrogliopathy, which underlies several neurodegenerative pathologies linked to cognitive deficits [79, 80]. In particular, age-dependent tau astrogliopathy was consistently observed in the brains of ‘super-centenarians’ aged more than 110 years [81]. Thus age-dependent cognitive decline is, at least in part, linked to neuroglial decline and paralysis.

## Glia-based enhancement of cognitive reserve: the role of the noradrenergic system

### Lifestyle

Lifestyle, which includes dieting, exercise, sociocultural and intellectual engagement, is known to be a powerful factor modifying cognitive reserve and cognitive longevity [82, 83]. Experiments on various animal models and on aged animals demonstrated that, at least in part, cognition-enhancing effects of environmental stimulation and physical exercise are mediated through an increase in neuroglial morphology and homeostatic support. Brain ageing as well as chronic brain diseases are often associated with atrophy and asthenia of astrocytes and microglia; these atrophic changes can be reversed by various forms of environmental stimulation and dieting [78, 84–88]. Incidentally, replacing old atrophic astrocytes and microglia with young ones rescues cognitive deficits in aged mice [89, 90]. Exposure to enriched environment and physical exercise also improves neurogenesis in AD mice [91]; similar effect is achieved through dietary supplementation with polyunsaturated fatty acid 2-hydroxy-docosahexaenoic acid [92]. Caloric restriction induces the growth of astroglial perisynaptic leaflets; consequent increase in synaptic coverage improves glutamate clearance, K^+^ buffering and translates into increased synaptic plasticity [93].

### Noradrenergic innervation

At the neurochemical level, effects of environmental stimulation and lifestyle on the brain are mediated, at least partially, through the noradrenergic pan-brain innervation mainly provided by neurones of the *Locus coeruleus* (LC), a small nucleus located at the fourth ventricle; LC is the main source (~70%) of noradrenaline (NA) in the CNS [94–96]. In ageing and chronic age-dependent pathologies LC neurones degenerate thus limiting noradrenergic bioavailability [97, 98], it was even suggested that the demise of the noradrenergic system may be a possible causative factor in AD [99, 100].

Astrocytes are primary cellular targets for NA through an abundant expression of α- and β-adrenoceptors [101]. Additionally, astrocytes are the sole possessors of MAO-B, the central enzyme of catecholaminergic catabolism. Expression of MAO-B is known to increase in ageing and neurodegeneration, which may further limit noradrenergic innervation [102]. Indeed, aberrant astrocytic Ca^2+^ signals due to the reduced levels of noradrenaline were found in mouse AD model [103]. Usage of MAO-B inhibitors, reversible and irreversible, may be a valid strategy to increase cognitive reserve [102]. Transcranial direct current stimulation (tDCS), known to improve memory, facilitate motor rehabilitation, alleviate depression, and slow down the progression of cognitive impairments in AD patients [104] acts through astrocytes and their adrenoceptors. Exposure to tDCS triggers massive astrocytic Ca^2+^ signals that are inhibited by the ablation of noradrenergic neurones or by pharmacological inhibition of α_1_-adrenoceptors [105, 106].

## Summary

Cognitive reserve reflects the life-long adaptation to the exposome that dynamically shapes the brain structure through functional use of the brain and contributes to the brain capacity to withstand the damage. Neuroglia, the principle homeostatic and defensive element of the CNS, play leading role in defining the cognitive reserve. Targeting neuroglia, both pharmacological and holistic, represents the novel strategy for improving the cognitive reserve and prolonging cognitive longevity.

## References

[1] Stern Y. What is cognitive reserve? Theory and research application of the reserve concept. J Int Neuropsychol Soc. 2002;8:448–60. 11939702

[2] Stern Y. Cognitive reserve. Neuropsychologia. 2009;47:2015–28. 19467352 10.1016/j.neuropsychologia.2009.03.004 PMC2739591

[3] Stern Y. Cognitive reserve in ageing and Alzheimer’s disease. Lancet Neurol. 2012;11:1006–12. 23079557 10.1016/S1474-4422(12)70191-6 PMC3507991

[4] Stern Y, Barnes CA, Grady C, Jones RN, Raz N. Brain reserve, cognitive reserve, compensation, and maintenance: operationalization, validity, and mechanisms of cognitive resilience. Neurobiol Aging. 2019;83:124–9. 31732015 10.1016/j.neurobiolaging.2019.03.022 PMC6859943

[5] Stern Y, Barulli D. Cognitive reserve. Handb Clin Neurol. 2019;167:181–90. 31753132 10.1016/B978-0-12-804766-8.00011-X

[6] Nelson PT, Alafuzoff I, Bigio EH, Bouras C, Braak H, Cairns NJ, et al. Correlation of Alzheimer disease neuropathologic changes with cognitive status: a review of the literature. J Neuropathol Exp Neurol. 2012;71:362–81. 22487856 10.1097/NEN.0b013e31825018f7 PMC3560290

[7] Nelson PT, Head E, Schmitt FA, Davis PR, Neltner JH, Jicha GA, et al. Alzheimer’s disease is not “brain aging”: neuropathological, genetic, and epidemiological human studies. Acta Neuropathol. 2011;121:571–87. 21516511 10.1007/s00401-011-0826-y PMC3179861

[8] Blessed G, Tomlinson BE, Roth M. The association between quantitative measures of dementia and of senile change in the cerebral grey matter of elderly subjects. Br J Psychiatry. 1968;114:797–811. 5662937 10.1192/bjp.114.512.797

[9] Ince PG. Pathological correlates of late-onset dementia in a multicentre, community-based population in England and Wales. Neuropathology Group of the Medical Research Council Cognitive Function and Ageing Study (MRC CFAS). Lancet. 2001;357:169–75. 11213093 10.1016/s0140-6736(00)03589-3

[10] Kittner SJ, White LR, Farmer ME, Wolz M, Kaplan E, Moes E, et al. Methodological issues in screening for dementia: the problem of education adjustment. J Chronic Dis. 1986;39:163–70. 3949940 10.1016/0021-9681(86)90019-6

[11] Zhang MY, Katzman R, Salmon D, Jin H, Cai GJ, Wang ZY, et al. The prevalence of dementia and Alzheimer’s disease in Shanghai, China: impact of age, gender, and education. Ann Neurol. 1990;27:428–37. 2353798 10.1002/ana.410270412

[12] Ahangari N, Fischer CE, Schweizer TA, Munoz DG. Cognitive resilience and severe Alzheimer’s disease neuropathology. Aging Brain. 2023;3:100065. 36911256 10.1016/j.nbas.2023.100065 PMC9997171

[13] Dai Q, Smith GD. Resilience to depression: implication for psychological vaccination. Front Psychiatry. 2023;14:1071859. 36865075 10.3389/fpsyt.2023.1071859 PMC9971009

[14] Anacker C, Scholz J, O’Donnell KJ, Allemang-Grand R, Diorio J, Bagot RC, et al. Neuroanatomic differences associated with stress susceptibility and resilience. Biol Psychiatry. 2016;79:840–9. 26422005 10.1016/j.biopsych.2015.08.009 PMC5885767

[15] Katzman R. Alzheimer’s disease as an age-dependent disorder. Ciba Found Symp. 1988;134:69–85. 2896109 10.1002/9780470513583.ch6

[16] Katzman R, Terry R, DeTeresa R, Brown T, Davies P, Fuld P, et al. Clinical, pathological, and neurochemical changes in dementia: a subgroup with preserved mental status and numerous neocortical plaques. Ann Neurol. 1988;23:138–44. 2897823 10.1002/ana.410230206

[17] Schofield PW, Logroscino G, Andrews HF, Albert S, Stern Y. An association between head circumference and Alzheimer’s disease in a population-based study of aging and dementia. Neurology. 1997;49:30–7. 9222166 10.1212/wnl.49.1.30

[18] Satz P, Morgenstern H, Miller EN, Selnes OA, McArthur JC, Cohen BA, et al. Low education as a possible risk factor for cognitive abnormalities in HIV-1: findings from the multicenter AIDS Cohort Study (MACS). J Acquir Immune Defic Syndr. 1993;6:503–11. 8483113

[19] Verkhratsky A, Butt AM. Neuroglia: function and pathology. New York: Elsevier; 2023.

[20] Verkhratsky A, Nedergaard M. The homeostatic astroglia emerges from evolutionary specialization of neural cells. Philos Trans R Soc Lond B Biol Sci. 2016;371:20150428. 27377722 10.1098/rstb.2015.0428 PMC4938028

[21] Semyanov A, Verkhratsky A. Astrocytic processes: from tripartite synapses to the active milieu. Trends Neurosci. 2021;44:781–92. 34479758 10.1016/j.tins.2021.07.006

[22] Vardjan N, Kreft M, Zorec R. Regulated exocytosis in astrocytes is as slow as the metabolic availability of gliotransmitters: focus on glutamate and ATP. Adv Neurobiol. 2014;11:81–101. 25236725 10.1007/978-3-319-08894-5_5

[23] Park DC, Bischof GN. The aging mind: neuroplasticity in response to cognitive training. Dialogues Clin Neurosci. 2013;15:109–19. 23576894 10.31887/DCNS.2013.15.1/dpark PMC3622463

[24] Zlokovic BV. Neurovascular mechanisms of Alzheimer’s neurodegeneration. Trends Neurosci. 2005;28:202–8. 15808355 10.1016/j.tins.2005.02.001

[25] Dias DO, Kim H, Holl D, Werne Solnestam B, Lundeberg J, Carlen M, et al. Reducing pericyte-derived scarring promotes recovery after spinal cord injury. Cell. 2018;173:153–65.e122. 29502968 10.1016/j.cell.2018.02.004 PMC5871719

[26] Escartin C, Galea E, Lakatos A, O’Callaghan JP, Petzold GC, Serrano-Pozo A, et al. Reactive astrocyte nomenclature, definitions, and future directions. Nat Neurosci. 2021;24:312–25. 33589835 10.1038/s41593-020-00783-4 PMC8007081

[27] Pekny M, Pekna M, Messing A, Steinhauser C, Lee JM, Parpura V, et al. Astrocytes: a central element in neurological diseases. Acta Neuropathol. 2016;131:323–45. 26671410 10.1007/s00401-015-1513-1

[28] Verkhratsky A, Butt A, Li B, Illes P, Zorec R, Semyanov A, et al. Astrocytes in human central nervous system diseases: a frontier for new therapies. Signal Transduct Target Ther. 2023;8:396. 37828019 10.1038/s41392-023-01628-9 PMC10570367

[29] Augusto-Oliveira M, Arrifano GP, Takeda PY, Lopes-Araujo A, Santos-Sacramento L, Anthony DC, et al. Astroglia-specific contributions to the regulation of synapses, cognition and behaviour. Neurosci Biobehav Rev. 2020;118:331–57. 32768488 10.1016/j.neubiorev.2020.07.039

[30] Kriegstein A, Alvarez-Buylla A. The glial nature of embryonic and adult neural stem cells. Annu Rev Neurosci. 2009;32:149–84. 19555289 10.1146/annurev.neuro.051508.135600 PMC3086722

[31] Yeh CY, Wu KY, Huang GJ, Verkhratsky A. Radial stem astrocytes (aka neural stem cells): Identity, development, physio-pathology, and therapeutic potential. Acta Physiol. 2023;238:e13967. 10.1111/apha.13967 36971751

[32] Gotz M, Nakafuku M, Petrik D. Neurogenesis in the developing and adult brain-similarities and key differences. Cold Spring Harb Perspect Biol. 2016;8:a018853. 27235475 10.1101/cshperspect.a018853 PMC4930921

[33] Allen NJ. Astrocyte regulation of synaptic behavior. Annu Rev Cell Dev Biol. 2014;30:439–63. 25288116 10.1146/annurev-cellbio-100913-013053

[34] Christopherson KS, Ullian EM, Stokes CC, Mullowney CE, Hell JW, Agah A, et al. Thrombospondins are astrocyte-secreted proteins that promote CNS synaptogenesis. Cell. 2005;120:421–33. 15707899 10.1016/j.cell.2004.12.020

[35] Pfrieger FW, Barres BA. Synaptic efficacy enhanced by glial cells in vitro. Science. 1997;277:1684–7. 9287225 10.1126/science.277.5332.1684

[36] Ullian EM, Sapperstein SK, Christopherson KS, Barres BA. Control of synapse number by glia. Science. 2001;291:657–61. 11158678 10.1126/science.291.5504.657

[37] Kucukdereli H, Allen NJ, Lee AT, Feng A, Ozlu MI, Conatser LM, et al. Control of excitatory CNS synaptogenesis by astrocyte-secreted proteins Hevin and SPARC. Proc Natl Acad Sci USA. 2011;108:E440–9. 21788491 10.1073/pnas.1104977108 PMC3156217

[38] Verkhratsky A, Matteoli M, Parpura V, Mothet JP, Zorec R. Astrocytes as secretory cells of the central nervous system: idiosyncrasies of vesicular secretion. EMBO J. 2016;35:239–57. 26758544 10.15252/embj.201592705 PMC4741299

[39] Kettenmann H, Kirchhoff F, Verkhratsky A. Microglia: new roles for the synaptic stripper. Neuron. 2013;77:10–18. 23312512 10.1016/j.neuron.2012.12.023

[40] Paolicelli RC, Bolasco G, Pagani F, Maggi L, Scianni M, Panzanelli P, et al. Synaptic pruning by microglia is necessary for normal brain development. Science. 2011;333:1456–8. 21778362 10.1126/science.1202529

[41] Tremblay ME, Lowery RL, Majewska AK. Microglial interactions with synapses are modulated by visual experience. PLoS Biol. 2010;8:e1000527. 21072242 10.1371/journal.pbio.1000527 PMC2970556

[42] Weinhard L, di Bartolomei G, Bolasco G, Machado P, Schieber NL, Neniskyte U, et al. Microglia remodel synapses by presynaptic trogocytosis and spine head filopodia induction. Nat Commun. 2018;9:1228. 29581545 10.1038/s41467-018-03566-5 PMC5964317

[43] Nave KA, Werner HB. Ensheathment and myelination of axons: evolution of glial functions. Annu Rev Neurosci. 2021;44:197–219. 33722070 10.1146/annurev-neuro-100120-122621

[44] de Faria O Jr., Gonsalvez DG, Nicholson M, Xiao J. Activity-dependent central nervous system myelination throughout life. J Neurochem. 2019;148:447–61. 30225984 10.1111/jnc.14592 PMC6587454

[45] Fields RD. A new mechanism of nervous system plasticity: activity-dependent myelination. Nat Rev Neurosci. 2015;16:756–67. 26585800 10.1038/nrn4023 PMC6310485

[46] Bernard C. Leçons sur les phénomènes de la vie communs aux animaux et aux végétaux. Paris: Bailliere; 1878.

[47] Cannon WB. Organization for physiological homeostasis. Physiol Rev. 1929;9:399–431.

[48] Davies KJ. Adaptive homeostasis. Mol Aspects Med. 2016;49:1–7. 27112802 10.1016/j.mam.2016.04.007 PMC4868097

[49] Sterling P, Eyer J. Allostasis: a new paradigm to explain arousal pathology. In: Fisher S, Reason J, editors. Handbook of life stress, cognition and health. Chichester: John Wiley & Sons; 1988. pp. 629–240.

[50] Untiet V, Beinlich FRM, Kusk P, Kang N, Ladron-de-Guevara A, Song W, et al. Astrocytic chloride is brain state dependent and modulates inhibitory neurotransmission in mice. Nat Commun. 2023;14:1871. 37015909 10.1038/s41467-023-37433-9 PMC10073105

[51] Rose CR, Verkhratsky A. Sodium homeostasis and signalling: the core and the hub of astrocyte function. Cell Calcium. 2024;117:102817. 37979342 10.1016/j.ceca.2023.102817

[52] MacAulay N. Molecular mechanisms of K^+^ clearance and extracellular space shrinkage-Glia cells as the stars. Glia. 2020;68:2192–211. 32181522 10.1002/glia.23824

[53] Li B, Yu W, Verkhratsky A. Trace metals and astrocytes physiology and pathophysiology. Cell Calcium. 2024;118:102843. 38199057 10.1016/j.ceca.2024.102843

[54] Dringen R, Gutterer JM, Hirrlinger J. Glutathione metabolism in brain metabolic interaction between astrocytes and neurons in the defense against reactive oxygen species. Eur J Biochem. 2000;267:4912–6. 10931173 10.1046/j.1432-1327.2000.01597.x

[55] Dringen R, Pfeiffer B, Hamprecht B. Synthesis of the antioxidant glutathione in neurons: supply by astrocytes of CysGly as precursor for neuronal glutathione. J Neurosci. 1999;19:562–9. 9880576 10.1523/JNEUROSCI.19-02-00562.1999 PMC6782200

[56] Makar TK, Nedergaard M, Preuss A, Gelbard AS, Perumal AS, Cooper AJ. Vitamin E, ascorbate, glutathione, glutathione disulfide, and enzymes of glutathione metabolism in cultures of chick astrocytes and neurons: evidence that astrocytes play an important role in antioxidative processes in the brain. J Neurochem. 1994;62:45–53. 7903354 10.1046/j.1471-4159.1994.62010045.x

[57] Pellerin L, Magistretti PJ. Sweet sixteen for ANLS. J Cereb Blood Flow Metab. 2012;32:1152–66. 22027938 10.1038/jcbfm.2011.149 PMC3390819

[58] Fink K, Velebit J, Vardjan N, Zorec R, Kreft M. Noradrenaline-induced l-lactate production requires d-glucose entry and transit through the glycogen shunt in single-cultured rat astrocytes. J Neurosci Res. 2021;99:1084–98. 33491223 10.1002/jnr.24783

[59] Rae CD, Baur JA, Borges K, Dienel G, Diaz-Garcia CM, Douglass SR, et al. Brain energy metabolism: a roadmap for future research. J Neurochem. 2024;168:910–54. 38183680 10.1111/jnc.16032 PMC11102343

[60] Simons M, Nave KA. Oligodendrocytes: myelination and axonal support. Cold Spring Harb Perspect Biol. 2015;8:a020479. 26101081 10.1101/cshperspect.a020479 PMC4691794

[61] Yi C, Verkhratsky A, Niu J. Pathological potential of oligodendrocyte precursor cells: terra incognita. Trends Neurosci. 2023;46:581–96. 37183154 10.1016/j.tins.2023.04.003

[62] Stratoulias V, Venero JL, Tremblay ME, Joseph B. Microglial subtypes: diversity within the microglial community. EMBO J. 2019;38:e101997. 31373067 10.15252/embj.2019101997 PMC6717890

[63] Aten S, Du Y, Taylor O, Dye C, Collins K, Thomas M, et al. Chronic stress impairs the structure and function of astrocyte networks in an animal model of depression. Neurochem Res. 2023;48:1191–210. 35796915 10.1007/s11064-022-03663-4 PMC9823156

[64] Lin SS, Zhou B, Chen BJ, Jiang RT, Li B, Illes P, et al. Electroacupuncture prevents astrocyte atrophy to alleviate depression. Cell Death Dis. 2023;14:343. 37248211 10.1038/s41419-023-05839-4 PMC10227075

[65] Sofroniew MV. Astrocyte reactivity: subtypes, states, and functions in CNS innate immunity. Trends Immunol. 2020;41:758–70. 32819810 10.1016/j.it.2020.07.004 PMC7484257

[66] Burda JE, O’Shea TM, Ao Y, Suresh KB, Wang S, Bernstein AM, et al. Divergent transcriptional regulation of astrocyte reactivity across disorders. Nature. 2022;606:557–64. 35614216 10.1038/s41586-022-04739-5 PMC10027402

[67] Mills WA 3rd, Woo AM, Jiang S, Martin J, Surendran D, Bergstresser M, et al. Astrocyte plasticity in mice ensures continued endfoot coverage of cerebral blood vessels following injury and declines with age. Nat Commun. 2022;13:1794. 35379828 10.1038/s41467-022-29475-2 PMC8980042

[68] O’Shea TM, Ao Y, Wang S, Wollenberg AL, Kim JH, Ramos Espinoza RA, et al. Lesion environments direct transplanted neural progenitors towards a wound repair astroglial phenotype in mice. Nat Commun. 2022;13:5702. 36171203 10.1038/s41467-022-33382-x PMC9519954

[69] Lindvall O, Kokaia Z. Neurogenesis following stroke affecting the adult brain. Cold Spring Harb Perspect Biol. 2015;7:a019034. 26525150 10.1101/cshperspect.a019034 PMC4632663

[70] Rahman AA, Amruta N, Pinteaux E, Bix GJ. Neurogenesis after stroke: a therapeutic perspective. Transl Stroke Res. 2021;12:1–14. 32862401 10.1007/s12975-020-00841-w PMC7803692

[71] Olabarria M, Noristani HN, Verkhratsky A, Rodriguez JJ. Concomitant astroglial atrophy and astrogliosis in a triple transgenic animal model of Alzheimer’s disease. Glia. 2010;58:831–8. 20140958 10.1002/glia.20967

[72] Condello C, Yuan P, Grutzendler J. Microglia-mediated neuroprotection, TREM2, and Alzheimer’s disease: evidence from optical imaging. Biol Psychiatry. 2018;83:377–87. 29169609 10.1016/j.biopsych.2017.10.007 PMC5767550

[73] Condello C, Yuan P, Schain A, Grutzendler J. Microglia constitute a barrier that prevents neurotoxic protofibrillar Ab42 hotspots around plaques. Nat Commun. 2015;6:6176. 25630253 10.1038/ncomms7176 PMC4311408

[74] Verkhratsky A, Marutle A, Rodriguez-Arellano JJ, Nordberg A. Glial asthenia and functional paralysis: a new perspective on neurodegeneration and Alzheimer’s disease. Neuroscientist. 2015;21:552–68. 25125026 10.1177/1073858414547132

[75] Pakkenberg B, Gundersen HJ. Neocortical neuron number in humans: effect of sex and age. J Comp Neurol. 1997;384:312–20. 9215725

[76] Marner L, Nyengaard JR, Tang Y, Pakkenberg B. Marked loss of myelinated nerve fibers in the human brain with age. J Comp Neurol. 2003;462:144–52. 12794739 10.1002/cne.10714

[77] Streit WJ, Sammons NW, Kuhns AJ, Sparks DL. Dystrophic microglia in the aging human brain. Glia. 2004;45:208–12. 14730714 10.1002/glia.10319

[78] Popov A, Brazhe N, Morozova K, Yashin K, Bychkov M, Nosova O, et al. Mitochondrial malfunction and atrophy of astrocytes in the aged human cerebral cortex. Nat Commun. 2023;14:8380. 38104196 10.1038/s41467-023-44192-0 PMC10725430

[79] Kovacs GG, Ferrer I, Grinberg LT, Alafuzoff I, Attems J, Budka H, et al. Aging-related tau astrogliopathy (ARTAG): harmonized evaluation strategy. Acta Neuropathol. 2016;131:87–102. 26659578 10.1007/s00401-015-1509-x PMC4879001

[80] Schultz C, Dehghani F, Hubbard GB, Thal DR, Struckhoff G, Braak E, et al. Filamentous tau pathology in nerve cells, astrocytes, and oligodendrocytes of aged baboons. J Neuropathol Exp Neurol. 2000;59:39–52. 10744034 10.1093/jnen/59.1.39

[81] Takao M, Hirose N, Arai Y, Mihara B, Mimura M. Neuropathology of supercentenarians - four autopsy case studies. Acta Neuropathol Commun. 2016;4:97. 27590044 10.1186/s40478-016-0368-6 PMC5010697

[82] Bredesen DE. Reversal of cognitive decline: a novel therapeutic program. Aging. 2014;6:707–17. 25324467 10.18632/aging.100690 PMC4221920

[83] Buchman AS, Boyle PA, Yu L, Shah RC, Wilson RS, Bennett DA. Total daily physical activity and the risk of AD and cognitive decline in older adults. Neurology. 2012;78:1323–9. 22517108 10.1212/WNL.0b013e3182535d35 PMC3335448

[84] Augusto-Oliveira M, Verkhratsky A. Mens sana in corpore sano: lifestyle changes modify astrocytes to contain Alzheimer’s disease. Neural Regen Res. 2021;16:1548–9. 33433476 10.4103/1673-5374.303023 PMC8323677

[85] Augusto-Oliveira M, Verkhratsky A. Lifestyle-dependent microglial plasticity: training the brain guardians. Biol Direct. 2021;16:12. 34353376 10.1186/s13062-021-00297-4 PMC8340437

[86] Verkhratsky A, Rodrigues JJ, Pivoriunas A, Zorec R, Semyanov A. Astroglial atrophy in Alzheimer’s disease. Pflugers Arch. 2019;471:1247–61. 31520182 10.1007/s00424-019-02310-2

[87] Rodriguez JJ, Terzieva S, Olabarria M, Lanza RG, Verkhratsky A. Enriched environment and physical activity reverse astrogliodegeneration in the hippocampus of AD transgenic mice. Cell Death Dis. 2013;4:e678. 23788035 10.1038/cddis.2013.194 PMC3702309

[88] Beauquis J, Pavia P, Pomilio C, Vinuesa A, Podlutskaya N, Galvan V, et al. Environmental enrichment prevents astroglial pathological changes in the hippocampus of APP transgenic mice, model of Alzheimer’s disease. Exp Neurol. 2013;239:28–37. 23022919 10.1016/j.expneurol.2012.09.009

[89] Yang Z, Gong M, Jian T, Li J, Yang C, Ma Q, et al. Engrafted glial progenitor cells yield long-term integration and sensory improvement in aged mice. Stem Cell Res Ther. 2022;13:285. 35765112 10.1186/s13287-022-02959-0 PMC9241208

[90] Elmore MRP, Hohsfield LA, Kramar EA, Soreq L, Lee RJ, Pham ST, et al. Replacement of microglia in the aged brain reverses cognitive, synaptic, and neuronal deficits in mice. Aging Cell. 2018;17:e12832. 30276955 10.1111/acel.12832 PMC6260908

[91] Rodriguez JJ, Noristani HN, Olabarria M, Fletcher J, Somerville TD, Yeh CY, et al. Voluntary running and environmental enrichment restores impaired hippocampal neurogenesis in a triple transgenic mouse model of Alzheimer’s disease. Curr Alzheimer Res. 2011;8:707–17. 21453244 10.2174/156720511797633214

[92] Fiol-deRoque MA, Gutierrez-Lanza R, Torres M, Terés S, Barceló P, Rial RV, et al. Cognitive recovery and restoration of cell proliferation in the dentate gyrus in the 5XFAD transgenic mice model of Alzheimer’s disease following 2-hydroxy-DHA treatment. Biogerontology. 2013;14:763–75. 24114505 10.1007/s10522-013-9461-4

[93] Popov A, Denisov P, Bychkov M, Brazhe A, Lyukmanova E, Shenkarev Z, et al. Caloric restriction triggers morphofunctional remodeling of astrocytes and enhances synaptic plasticity in the mouse hippocampus. Cell Death Dis. 2020;11:208. 32231202 10.1038/s41419-020-2406-3 PMC7105492

[94] Feinstein DL, Kalinin S, Braun D. Causes, consequences, and cures for neuroinflammation mediated via the locus coeruleus: noradrenergic signaling system. J Neurochem. 2016;139:154–78. 26968403 10.1111/jnc.13447

[95] Foote SL, Bloom FE, Aston-Jones G. Nucleus locus ceruleus: new evidence of anatomical and physiological specificity. Physiol Rev. 1983;63:844–914. 6308694 10.1152/physrev.1983.63.3.844

[96] Marien MR, Colpaert FC, Rosenquist AC. Noradrenergic mechanisms in neurodegenerative diseases: a theory. Brain Res Brain Res Rev. 2004;45:38–78. 15063099 10.1016/j.brainresrev.2004.02.002

[97] Wilson RS, Nag S, Boyle PA, Hizel LP, Yu L, Buchman AS, et al. Neural reserve, neuronal density in the locus ceruleus, and cognitive decline. Neurology. 2013;80:1202–8. 23486878 10.1212/WNL.0b013e3182897103 PMC3691778

[98] Leanza G, Gulino R, Zorec R. Noradrenergic hypothesis linking neurodegeneration-based cognitive decline and astroglia. Front Mol Neurosci. 2018;11:254. 30100866 10.3389/fnmol.2018.00254 PMC6072880

[99] Robertson IH. A noradrenergic theory of cognitive reserve: implications for Alzheimer’s disease. Neurobiol Aging. 2013;34:298–308. 22743090 10.1016/j.neurobiolaging.2012.05.019

[100] Slater C, Wang Q. Alzheimer’s disease: an evolving understanding of noradrenergic involvement and the promising future of electroceutical therapies. Clin Transl Med. 2021;11:e397. 33931975 10.1002/ctm2.397 PMC8087948

[101] Verkhratsky A, Nedergaard M. Physiology of astroglia. Physiol Rev. 2018;98:239–389. 29351512 10.1152/physrev.00042.2016 PMC6050349

[102] Park JH, Ju YH, Choi JW, Song HJ, Jang BK, Woo J, et al. Newly developed reversible MAO-B inhibitor circumvents the shortcomings of irreversible inhibitors in Alzheimer’s disease. Sci Adv. 2019;5:eaav0316. 30906861 10.1126/sciadv.aav0316 PMC6426469

[103] Abjorsbraten KS, Skaaraas G, Cunen C, Bjornstad DM, Binder KMG, Bojarskaite L, et al. Impaired astrocytic Ca^2+^ signaling in awake-behaving Alzheimer’s disease transgenic mice. Elife. 2022;11:e75055. 35833623 10.7554/eLife.75055 PMC9352348

[104] Kuo MF, Paulus W, Nitsche MA. Therapeutic effects of non-invasive brain stimulation with direct currents (tDCS) in neuropsychiatric diseases. Neuroimage. 2014;85:948–60. 23747962 10.1016/j.neuroimage.2013.05.117

[105] Monai H, Ohkura M, Tanaka M, Oe Y, Konno A, Hirai H, et al. Calcium imaging reveals glial involvement in transcranial direct current stimulation-induced plasticity in mouse brain. Nat Commun. 2016;7:11100. 27000523 10.1038/ncomms11100 PMC4804173

[106] Monai H, Hirase H. Astrocytes as a target of transcranial direct current stimulation (tDCS) to treat depression. Neurosci Res. 2018;126:15–21. 29079367 10.1016/j.neures.2017.08.012

